# Hyperbaric Oxygen Therapy Counters Oxidative Stress/Inflammation-Driven Symptoms in Long COVID-19 Patients: Preliminary Outcomes

**DOI:** 10.3390/metabo13101032

**Published:** 2023-09-25

**Authors:** Simona Mrakic-Sposta, Alessandra Vezzoli, Giacomo Garetto, Matteo Paganini, Enrico Camporesi, Tommaso Antonio Giacon, Cinzia Dellanoce, Jacopo Agrimi, Gerardo Bosco

**Affiliations:** 1 Institute of Clinical Physiology, National Research Council (IFC-CNR), 20162 Milan, Italy; cinziacarla.dellanoce@cnr.it; 2 ATIP Center for Hyperbaric Medicine, 35128 Padova, Italy; giacomo.garetto@mediclinic.it; 3 Department of Biomedical Sciences, University of Padova, 35122 Padova, Italy; matteo.paganini@unipd.it (M.P.); ecampore@usf.edu (E.C.); tommasoantonio.giacon@studenti.unipd.it (T.A.G.); jacopo.agrimi@unipd.it (J.A.); gerardo.bosco@unipd.it (G.B.)

**Keywords:** long COVID-19, HBOT, oxidative stress, reactive oxygen species, inflammation, saliva, urine, non-invasive methods, Electron Paramagnetic Resonance

## Abstract

Long COVID-19 patients show systemic inflammation and persistent symptoms such as fatigue and malaise, profoundly affecting their quality of life. Since improving oxygenation can oppose inflammation at multiple tissue levels, we hypothesized that hyperbaric oxygen therapy (HBOT) could arrest inflammation progression and thus relieve symptoms of COVID-19. We evaluated oxy-inflammation biomarkers in long COVID-19 subjects treated with HBOT and monitored with non-invasive methods. Five subjects (two athletes and three patients with other comorbidities) were assigned to receive HBOT: 100% inspired O_2_ at 2.4 ATA in a multiplace hyperbaric chamber for 90 min (three athletes: 15 HBOT × 5 days/wk for 3 weeks; two patients affected by Idiopathic Sudden Sensorineural Hearing Loss: 30 HBOT × 5 days/wk for 6 weeks; and one patient with osteomyelitis: 30 HBOT × 5 days/wk for week for 6 weeks and, after a 30-day break, followed by a second cycle of 20 HBOT). Using saliva and/or urine samples, reactive oxygen species (ROS), antioxidant capacity, cytokines, lipids peroxidation, DNA damage, and renal status were assessed at T1_pre (basal level) and at T2_pre (basal level after treatment), and the results showed attenuated ROS production, lipid peroxidation, DNA damage, NO metabolites, and inflammation biomarker levels, especially in the athletes post-treatment. Thus, HBOT may represent an alternative non-invasive method for treating long COVID-19-induced long-lasting manifestations of oxy-inflammation.

## 1. Introduction

COVID-19 manifests with different clinical features, ranging from asymptomatic infection to severe acute respiratory syndrome [1] or a multi-organ syndrome [2]. Of relevance, after the initial acute phase, up to 30% of non-hospitalized and 70% of hospitalized subjects develop unexpected sub-acute symptoms [3]. Among these long-lasting manifestations, five have been most commonly recognized: dyspnea (24%), hair loss (25%), cognitive impairment (27%), headache (44%), and fatigue (58%) [4]. Other symptoms include insomnia, loss of taste and smell, diffuse myalgias, and joint and musculoskeletal pain [5].

Both the acute disease and the “long COVID-19s” [6] are characterized by a “cytokines storm”, liable for systemic inflammation with higher levels of interleukin (IL) 6, 8, 10, 18 and Chemokine (C-C motif) ligand 5 (CCL5) [7,8,9,10,11,12]. Moreover, significantly higher IL-6 levels have been observed in severe cases compared to moderate cases [13]. In turn, a state of hyper-inflammation or immune dysfunction is often coupled with increased oxidative stress resulting from excessive reactive oxygen species (ROS) production and depleted antioxidant systems, ultimately damaging multiple substrates, such as cellular proteins, lipids, and nucleic acids [14,15,16].

Hyperbaric oxygen therapy (HBOT) is used in pathologies where tissues are starved for oxygen. The combined action of hyperoxia and hyperbaric pressure leads to a significant improvement in tissue oxygenation while targeting both O_2_ and pressure-sensitive genes [17,18]. The Undersea and Hyperbaric Medical Society (UHMS) describes HBOT as an intervention whereby patients breath near 100% oxygen while being pressurized to at least 1.4 to 2.5 atmosphere absolute (ATA) in a hyperbaric chamber [19]. Fourteen medical applications have been accepted by UHMS [20]. Recent clinical and preclinical results have suggested the potential usefulness of HBOT in treating long COVID-19 [21,22,23,24,25,26,27,28,29,30,31]. In particular, in neurological tissues, Zilberman-Itskovich et al. (2022) [32] provided some interesting observations about the effects of hyperbaric therapy on perfusion; in myocardial function, Leitman et al. (2023) [27] suggested that HBOT can indicate subclinical left ventricular dysfunction. HBOT has also yielded improvements in health, performance parameters, and in specific blood gas parameters, as reported by Kitala et al. (2023) [32].

The current pilot study aimed to investigate the effects of HBOT on oxy-inflammation biomarkers in non-invasively obtainable biofluids (saliva and urine) for the first time in the literature by investigating five subjects suffering from long COVID-19 (with or without other morbidities) with ongoing symptoms three months after confirmed infection.

## 2. Materials and Methods

### 2.1. Subjects

This pilot study involved five subjects who were diagnosed with COVID-19 in January–February 2021 (two professional athletes and three patients). Table 1 describes the included subjects’ features (evaluated at the time of the first visit, before the HBOT sessions). The athletes and ISHHL patients did not receive any medications or supplements from 2 weeks before and during the HBOT sessions, while a patient with osteomyelitis to the thumb received only nonsteroidal anti-inflammatory drugs when necessary.

All subjects had no history of allergic rhinitis and did not report any drug or food allergies. No alcohol consumption or smoking was reported. Based on the criteria for classifying the post-COVID-19 syndrome [31,33,34,35], three expert clinicians classified the five included subjects, as shown in Table 2. This proposed criterion is based on the initial symptoms, duration of symptoms, period of quiescence, and time of onset of symptoms. Five categories of post-COVID-19s: Type 1 includes duration of recovery (relates to the severity of infection), organ damage, and underlying medical conditions. Type 2 is characterized by symptoms persisting for six weeks from the onset of illness. Type 3 shows a period of quiescence or nearly full recovery after initial infection, followed by a recurrence of symptoms that persist for at least three months (Type 3A) or at least six months (Type 3B). Type 4 includes subjects who are initially asymptomatic at the time of a positive COVID-19 test but develop symptoms one to three months (Type 4A) or at least three months later (Type 4B) that persist for varying lengths of time. Type 5 includes those who have no or few symptoms at the time of a positive COVID-19 test and experience sudden death within the next 12 months [35].

The protocol of the study was approved by the Human Ethical Committee (HEC-DSB/04-19) of the Department of Biomedical Science of the University of Padova (Italy), and all subjects provided informed consent. The study was carried out according to the Declaration of Helsinki.

### 2.2. HBOT Protocol

**Figure 1:**
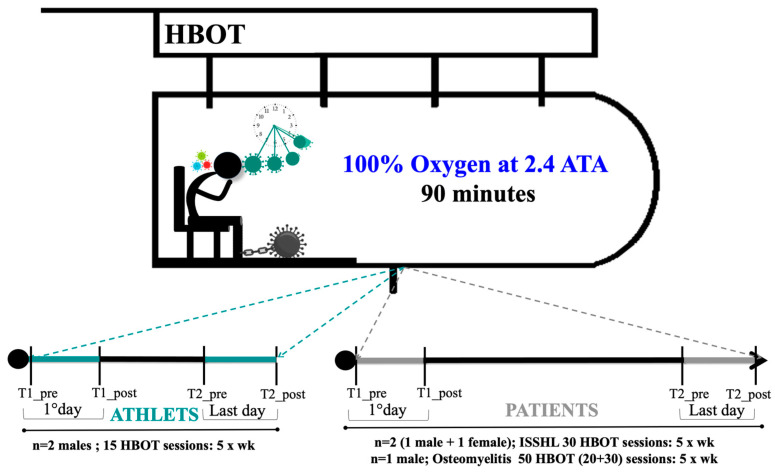
HBOT Experimental study design of working protocol with timeline of sample collection.

The five examined subjects were exposed to 100% inspired oxygen at 2.4 ATA in a multiplace hyperbaric chamber for 90 min using an overboard demand regulator and oral-nasal mask. The two athletes received 15 treatments of HBOT (5 days per week for 3 weeks). The two patients affected by ISSHL received 30 HBO treatments (5 days per week for 6 weeks); the patient with osteomyelitis received 30 HBO treatments (5 days per week for 6 weeks), and after a 30-day break, a second cycle of 20 HBOT sessions was administered [17] (Figure 1).

### 2.3. Fatigue Severity Scale

Fatigue was assessed according to a numeric rating scale (NRS) [36,37]. Subjects were asked to measure their average severity of fatigue over the last 24 h on a scale from 0 to 10 (0 = none; 10 = worst).

### 2.4. Saliva and Urine Samples Collection

Saliva and urine were collected on the 1st day of the HBOT session (T1_pre = basal value; T1_post = value after the 1st session) and on the final day of HBOT (T2_pre = basal value last day; T2_post = value after the last session HBOT) (see Figure 1). For saliva sampling, the subjects were instructed to refrain from drinking, eating, smoking, brushing their teeth, and using a mouthwash during the 30 min before salivary collection. They were instructed on the correct use of Salivette devices (Sarstedt, Nümbrecht, Germany). The samples were spun down, and approximately 1 mL of saliva was obtained [38,39,40,41]. Urine samples were collected via voluntary voiding in a sterile container provided to the subjects. All samples were stored in multiple aliquots at −80 °C until assayed and thawed only once before analysis.

The saliva samples were collected to determine ROS levels, total antioxidant capacity (TAC), and cytokine (IL-6, IL-1β, and TNF-α) concentrations. Lipid peroxidation (8-iso-pGF2α), DNA damage (8-OH-dG), Nitric Oxide metabolites (NO_2_ + NO_3_ = NOx), neopterin, creatinine, and uric acid concentrations were assessed based on the urine samples.

### 2.5. ROS by Electron Paramagnetic Resonance (EPR)

X-band (9.3 GHz) EPR spectroscopy, (E-Scan Bruker, Billerica, MA, USA) was used to detect ROS production in saliva as previously described [38,39,40,41]. Spin probe CMH (1-hydroxy-3-methoxycarbonyl-2,2,5,5-tetramethylpyrrolidine) was used to assess ROS, with CP• (3-Carboxy-2,2,5,5-tetramethyl-1-pyrrolidinyloxy), a stable radical, being used as an external reference. All samples were stabilized at 37 °C using Temperature Controller “Bio III” (Noxigen Science Transfer & Diagnostics GmbH, Elzach, Germany), along with a spectrometer. Spectra were recorded and analyzed using the Win EPR software (2.11 version) (supplied by Bruker).

### 2.6. Total Antioxidant Capacity (TAC)

The 6-hydroxy-2,5,7,8-tetramethylchroman-2-carboxylic acid (Trolox)-equivalent antioxidant capacity assay, a widely used kit-based commercial method, (Cayman Chemical, Ann Arbor, MI, USA, Item No. 709001) was used to assess the TAC levels as previously described [42,43,44,45,46,47]. Briefly, this assay is based on the ability of the antioxidants present in saliva to inhibit the oxidation of 2,2-azino-bis (3-ethylbenzothiazoline-6-sulfonic acid) (ABTS) to the radical cation (ABTS+) by a peroxidase; the antioxidant concentration is proportional to the absorbance signal suppression. The samples were read at 750 nm using a spectrophotometer. A linear calibration curve was computed from pure Trolox (6-hydroxy-2,5,7,8-tetramethylchroman-2-carboxylic acid)-containing reactions. TAC was expressed as trolox equivalent antioxidant capacity concentration (mM).

### 2.7. 8-Isoprostane (8-iso-PGF2α)

8-iso-PGF2α has been established as a marker of lipid peroxidation. A commercial competitive immunoassay of 8-isoprostane (Cayman Chemical, Ann Arbor, MI, USA, Item No. 516351) was used to assess the levels of 8-iso-PGF2α in urine as previously described [38,46,47,48,49,50]. The EIA employed an 8-iso-PGF2α tracer and an 8-iso-PGF2α antiserum. The sample 8-iso-PGF2α concentration was determined using a standard curve. The samples were read spectrophotometrically at a wavelength of 412 nm. 

### 2.8. 8-Hydroxy-2′-deoxyguanosine (8-OH-dG)

8-OH-dG has been established as a marker of nuclear oxidative DNA damage. A commercial ELISA kit (Cayman Chemical, Ann Arbor, MI, USA, Item No. 89320) was utilized to measure urinary concentrations of 8-OH-dG. The EIA employed an anti-mouse IgG-coated plate and a tracer consisting of an 8-OH-dG-enzyme conjugate, and the sample 8-OH-dG concentration was determined. The samples were read at a wavelength 412 nm, and the sample 8-OH-dG concentration was determined using an 8-OH-dG standard curve as previously described [45,46,49,51].

### 2.9. NO Metabolites (Nitrite and Nitrate)

Nitrite plus nitrate (NO_2_ + NO_3_ = NOx) concentration was assessed in urine via the use of a colorimetric method based on the Griess reaction [40,51,52] using a commercial kit (Cayman Chemical, Ann Arbor, MI, USA; Item No. 780001) that provided an accurate and convenient method for the measurement of nitrite and nitrate concentration. NO metabolites were determined at 545 nm. A linear calibration curve was computed from pure nitrate standard.

### 2.10. Quantification of Inflammatory Markers Levels in Saliva

IL-6, TNF-α, and IL-1β, saliva levels were determined by using ultrasensitive ELISA immunoassays (R&D Systems, Minneapolis, MN, USA) according to the manufacturer’s instructions [53,54,55]. The assays were based on a double-antibody sandwich technique. The levels of inflammatory markers (in pg/mL) in the saliva samples were calculated. Sample concentrations were determined for IL-6 at 450 nm and for TNF-α and IL-1β at 412 nm.

All samples were determined in duplicate using a microplate reader spectrophotometer (Infinite M200, Tecan Group Ltd., Männedorf, Switzerland).

### 2.11. Creatinine, Neopterin, and Uric Acid Concentration in Urine

Creatinine, neopterin, and uric acid concentrations were measured via the use of an isocratic high-pressure liquid chromatography (HPLC) method that has been previously described [39,40,47,50,56]. The concentration levels were measured using a Varian pump (240, auto sampler ProStar 410) coupled to a specific fluorometric detector. The calibration curves were linear over the range of 0.125–1 μmol/L for neopterin, 0.625–20 mmol/L for uric acid, and 1.25–10 mmol/L for the creatinine levels. The inter-assay and intra-assay coefficients of variation were <5%. 

### 2.12. Secondary Outcomes

In all subjects, standardized clinical hematological analyses were conducted by using an automated hematology analyzer according to the standard analysis methods of Azienda Ospedaliera of Padua laboratories.

### 2.13. Spirometry

We verified the impact of long COVID-19 on daily activity, focusing on lung function, via the use of traditional spirometry. Each subject underwent a clinical evaluation before HBOT. Furthermore, the 2 athletes repeated the spirometry at the end of the treatments. The spirometry parameters were recorded by using the turbine sensor of a portable spirometer (Pony FX; Cosmed; Rome, Italy) [49] with international standard: flowmeter, bidirectional digital turbine Ø 28 mm; flow range 0.08–20 L/s; volume range 12 L; accuracy of reading ±2%; resistance <0.6 cmH_2_O/L/s; temperature sensor 0–50 °C. We measured forced expiratory volume (FVC) in the first second (FEV1) FEV1/FVC%; peak expiratory flow (PEF); forced expiratory flow (FEF25-75%); and maximal expiratory flow (MEF) in the usual intervals (MEF75%, MEF50%, MEF25%). The equipment received the flow data in real-time at a frequency of 100 Hz before recording and exporting the data to a computer. The spirometry data were compared with predicted values, which were estimated on the basis of recent standards corrected for age and height.

### 2.14. Statistical Analysis

Data are presented as mean ± standard deviation (SD). Considering the low number of subjects reported, we performed a detailed statistical analysis. Percentage changes (Δ%), defined as [(postHBOT − preHBOT)/pre-HBOT) × 100], were used for the analysis to assess the effects of HBOT on the examined biomarkers. Also, an ANOVA with repeated measures and Dunn’s multiple comparison test were performed to further check the inter-group significance. dCohen was used to calculate the size effect, and a Confidence Interval 95% for dCohen was calculated. A *p* < 0.05 was considered statistically significant. Our statistical analysis was performed using SPSS statistics software (Version 25; IBM Corporation, Armonk, NY, USA) and the GraphPad Prism package for Mac (GraphPad Prism 9.5.1, GraphPad Software Inc., San Diego, CA, USA). 

## 3. Results

All the included subjects completed the study without reporting HBOT-related complications or discomfort.

### 3.1. Biomarker Oxy-Inflammation

**Figure 2:**
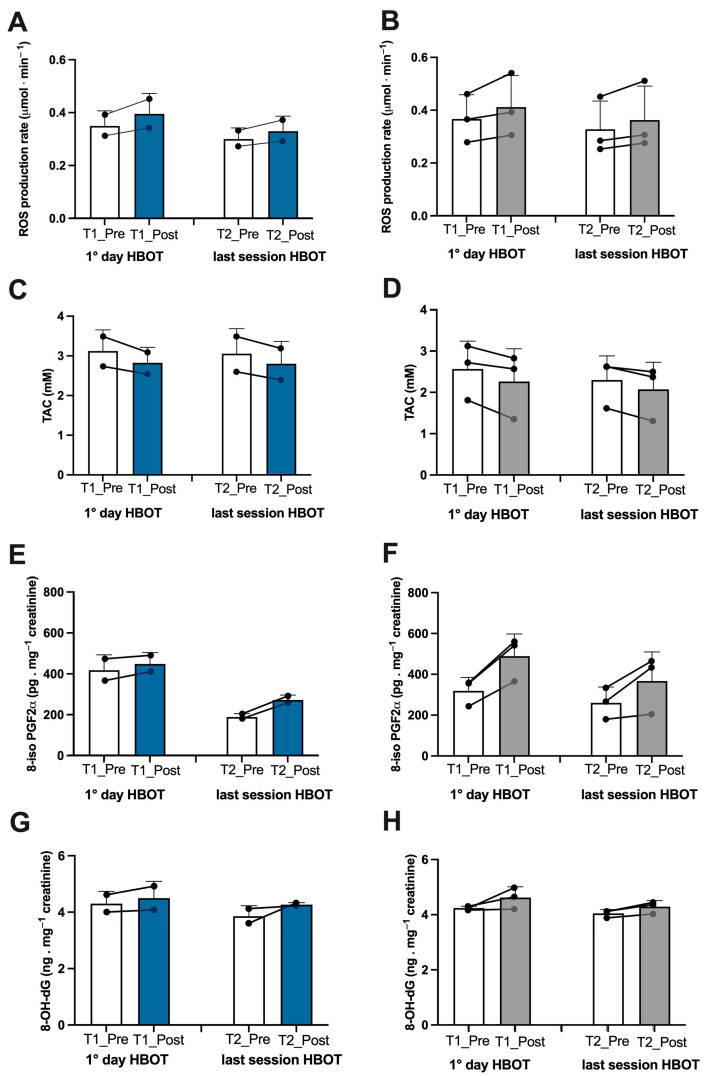
Effect of HBOT on oxidative stress in athletes and patients. Histogram plot (mean ± SD) and single plot of (**A**) ROS production (μmol.min^−^^1^), (**C**) antioxidant capacity (TAC), (**E**) lipid peroxidation (8-iso PGF2α), and (**G**) DNA oxidation (8-OH-dG) in athletes (white and blue bars) and (**B**,**D**,**F**,**H**) patients (white and grey bars).

**Figure 3:**
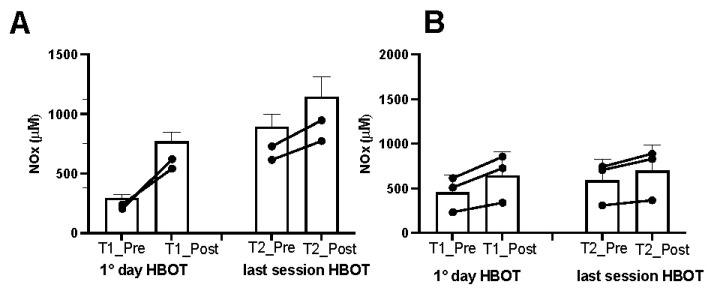
Effect of HBOT on NO metabolites (NOx) in (**A**) athletes (white and blue bars) and (**B**) patients (white and grey bars). Data are mean ± SD.

**Figure 4:**
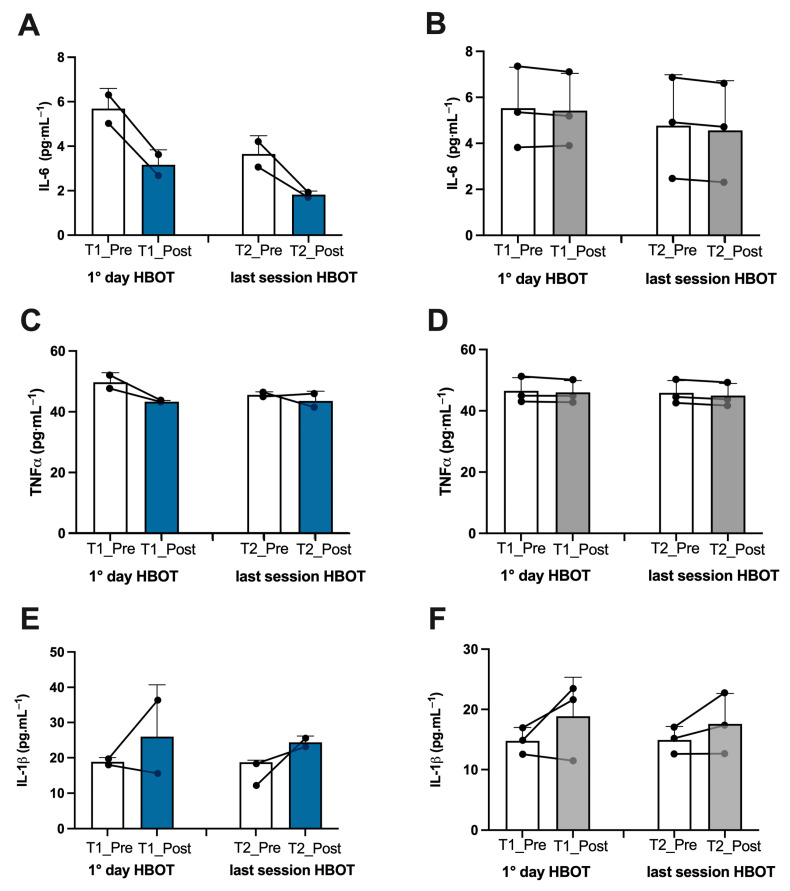
Effect of HBOT on inflammation in athletes and patients. Histogram plot (mean ± SD) and single plot of (**A**) IL-6, (**C**) TNF-α, and (**E**) IL-1β in athletes (white and blue bars) and (**B**,**D**,**F**) patients (white and gray bars).

**Figure 5:**
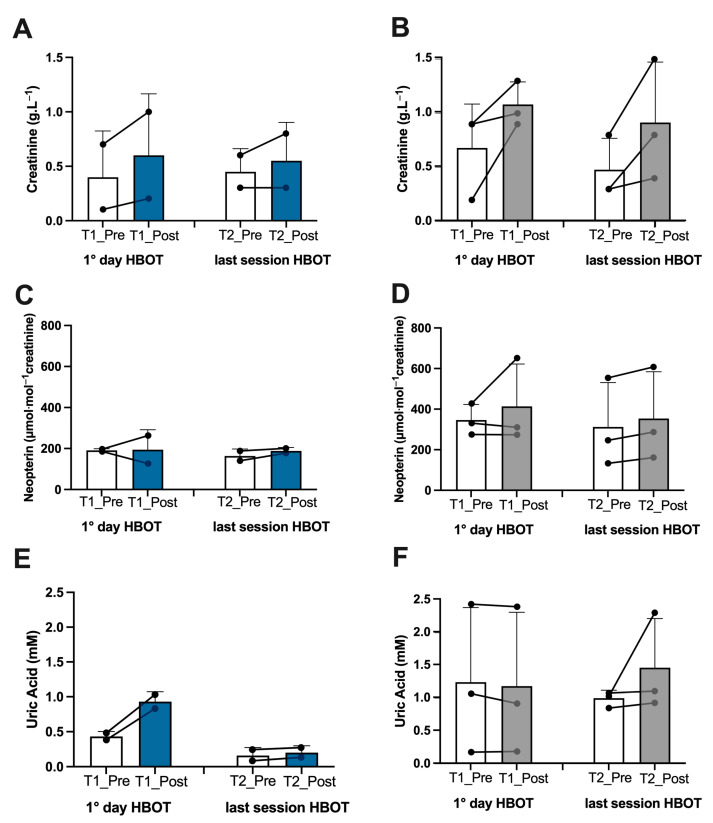
Effect of HBOT on renal function in athletes and patients. Histogram plot (mean ± SD) and single plot of (**A**) creatinine, (**C**) neopterin, and (**E**) uric Acid in athletes (white and blue bars) and (**B**,**D**,**F**) patients (white and gray bars).

Athletes. In the athletes, on the first day after treatment, we found an increase in ROS production (+13%), lipid peroxidation (+7%), DNA oxidation (+4.5%), and NO metabolite concentration (+252%), which was observed in tandem with a decline in antioxidant capacity (−10%) (Figure 2A,C,E,G, and Figure 3A). Inflammatory cytokines decreased—IL-6 (−44%) and TNFα (−13%) (Figure 4A,C). However, IL-1β increased by (+37%) (Figure 4E). Biomarkers of renal function were higher than at baseline—creatinine (+50%), neopterin (+2%), and uric acid (+116%) (Figure 5A,C,E). At the end of all sessions, after the last treatment (T2_Post vs. T2_Pre), we observed an increase in ROS production (+10%), lipid peroxidation (+44%), DNA oxidation (+11%), and NO metabolites (+422%). These changes were accompanied by a decrease in antioxidant capacity (−8%) (Figure 2A,C,E,G and Figure 3A). The circulating levels of inflammatory cytokines such as IL-6 and TNFα declined by 12% and 4%, respectively. Conversely, IL-1β increased by 30% (Figure 4A,C,E). Biomarkers of renal function increased—creatinine (+22%); neopterin (+15%); uric acid (+25%) (Figure 5A,C,E).

Non-athlete patients. In the patients, on the first day after treatment, we found an increase in ROS production (+13%), lipid peroxidation (+53%), DNA oxidation (+9%), and NO metabolite concentration (+35%) and a decrease in antioxidant capacity (−12%) (Figure 2B,D,F,H and Figure 3B). We observed a decline in the inflammatory cytokines IL-6 (−2%) and TNFα (−2%), along with an increase in IL-1β (+28%) (Figure 4B,D,F). Biomarkers of renal function increased—creatinine (+110%); neopterin (+20%). Uric acid decreased (−5%) (Figure 5B,D,F). At the end of the last session of treatment, we noticed an increase in ROS production (+11%), lipid peroxidation (+41%), DNA oxidation (+6%), and NO metabolites (+14%) and a decrease in antioxidant capacity (−10%) (Figure 2B,D,F,H and Figure 3B). Inflammatory cytokines decreased—IL-6 (−4%); TNFα (−2%). In stark contrast, IL-1β increased by +17% (Figure 4B,D,E). Biomarkers of renal function increased—creatinine (+93%); neopterin (+13%); uric acid (+47%) (Figure 5B,D,F).

Considering the basal levels recorded on the first day and on the last day before treatment (T1_Pre vs. T2_Pre), the athletes showed a decrease in ROS production (−14%), TAC (−2%), lipid peroxidation (−55%), DNA oxidation (−10%) (Figure 2A,C,E,G), NO metabolites (−205%) (Figure 3A), IL-6 (−36%), TNFα (−8%) (Figure 4A,C), neopterin (−14%), and uric acid (−62%) (Figure 5C,E) and an increase in creatinine (+12%) (Figure 5A).

Also, the patients showed a decrease in ROS production (−11%), TAC (−11%), lipid peroxidation (−18%), DNA oxidation (−5%), (Figure 2B,D,F,H), NO metabolites (−36%), (Figure 3B), IL-6 (−14%), (Figure 4B), creatinine (−30%), neopterin (−10%), and uric acid (−19%) (Figure 5B,D,F). No changes in TNFα and IL-1β (see Figure 4E,F) among the patients were recorded.

**Figure 6:**
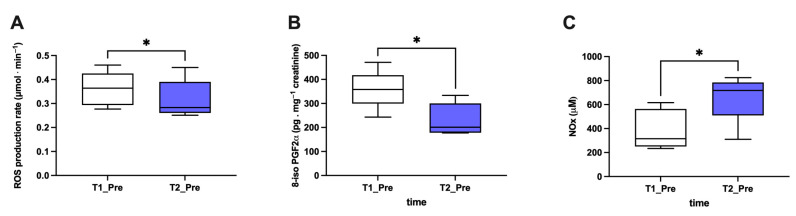
Significant effect of HBOT on biomarkers in all examined subjects. Histogram plot (mean ± SD) of (**A**) ROS production, (**B**) 8-iso PGF2α, and (**C**) NOx (collected at T1 and T2 pre-HBOT treatments). * *p* < 0.05 indicated a significant difference.

Finally, considering the subjects together (Figure 6), an overall significant reduction (*p* < 0.05) in ROS production (A; −14%; dCohen: 0.71) and lipid peroxidation (B; −35%; dCohen: 1.70) and an increase in NO metabolite (C; +70%; dCohen: 1.47) levels from T1_Pre and T2_Pre was observed.

The fatigue scores on the numerical rating scale (NRS) at T1_Pre were 7 ± 2.8 in athletes and 4.6 ± 3.3 in patients; at T2_Pre 2.0, the same metrics were ± 1.4 and 4.1 ± 4.1, respectively.

### 3.2. Hematological and Biochemical Analysis

The results derived from conducting hematological and biochemical tests on both athletes showed critical immunological profile changes in total lymphocytes and T cell compartment alterations, along with an increased expression of CD57+ in CD8 T cells. Moreover, high levels of mycoplasma pneumoniae (IgG, indicating a possible co/sub-infection between COVID-19 and atypical bacteria such as Mycoplasma pneumoniae) and liver enzyme abnormalities with aspartate aminotransferase (AST) were found. The ISSHL patients had hematological and biochemical results within the normal range, while in the patient affected by osteomyelitis, our hematological biochemical analysis results showed an increase in velocity sedimentation rate (VES) and C-reactive proteins (CRPs); these values remained altered even after HBOT.

### 3.3. Spirometry

The subjects were divided into athletes and patients. At basal examination, the athletes showed impaired lung function, specifically showing a reduction in lung volume (FVC—forced vital capacity: ~3.3; FEV1—forced expiratory volume in the first second: about 81%; FEV1/FVC% ~76.5%). After HBOT, improved lung function was recorded in both athletes, with a return to baseline values. In contrast, the patients showed normal basal spirometry, with no changes occurring after HBOT.

## 4. Discussion

To the best of our knowledge, this is the first study to monitor the oxidative stress, inflammation status, and immunological levels of subjects affected by long COVID-19, treated with HBOT, and examined via non-invasive methods (only saliva and urine were collected for the measurements). From this study, we aimed to gain a detailed insight into the physiological mechanisms involved in the above. None of the patients experienced any adverse reactions or complained of HBOT-related discomfort.

Recently, it has been reported that oxidative stress is likely implicated in the pathophysiology of all factors causing long COVID-19 and its symptoms [57] and that inflammation and oxidative stress mutually reinforce one another, thus contributing to the systemic hyperinflammatory state [58]. Furthermore, it is also worth mentioning how these responses might result in some alterations in physiological parameters, such as altered brain perfusion and metabolism [59,60], as a result of possible autonomic dysregulation and vascular damage [61].

The findings of this study show that ROS and oxidative damage to lipids and DNA are significant factors in long COVID-19 patients.

Also, our data confirm that the evolution of long COVID-19 is driven by cytokines produced in the body due to inflammation [62], which are often generated in response to viral infections and lead to oxidative stress, with immune status and altered lung function measures.

Experimental evidence shows that HBOT reduces the inflammation in animal models of sepsis (i.e., characterized by an overwhelming inflammatory response), with significant improvement in survival after a single treatment (2.4 ATA × 60 min) [63]. Additionally, HBOT could potentially mitigate oxidative stress [16,64] and inflammatory responses by reducing inflammatory cytokines through several transcriptional factors (i.e HIF-1 and NfKb) [65,66] and/or directly affecting the innate immune system [67].

HBOT stimulates the modulation of oxygen-sensitive transcription factors and ROS-mediated signaling pathways; in fact, our results show an increase in ROS production levels immediately after hyperbaric oxygen treatment (T1_post and T2_post) due to the hyperoxia exposition. Despite this, this increase in ROS subsided after subsequent treatment sessions, and at the end of HBOT, ROS levels decreased. One possible explanation for this is that the first HBOT cycle exerts a preconditioning activity by enhancing cellular protection against subsequent oxidative stress damage. In fact, we observed the same behavior with respect to the membrane lipids and DNA oxidation.

Recent studies show that an abnormal diffuse inflammatory cytokine profile can persist in long COVID-19 subjects for at least 8 months [68], along with the persistent deregulation of IL-1β, IL-6, and TNF-α [69]. Recent evidence links elevated cytokines to various symptoms in long COVID-19, including the peripheral immune response, neuroinflammation and microglial cell dysregulation, autonomic nervous system dysfunction, neuropathic pain, extreme fatigue, gastrointestinal tract symptoms, and arthralgia [70]. Furthermore, oxidative stress is another factor that contributes to cytokine release syndrome, and the signaling link between oxidative stress (OxS) and cytokines involves the p38 MAPK [71].

Therefore, HBOT assists in returning cytokines to homeostatic levels by modulating the interaction of IL-6 and/or TNFα, as our data proves. The response to hyperbaric oxygen therapy had an evident effect in the two young athletes in our study, both of whom had no comorbidities, and in the two patients in our patients, among whom the pathology underlying long COVID-19 was idiopathic sudden sensorineural hearing loss, a pathology that is considered to be an otolaryngologic emergency at rapid onset [72]. Unfortunately, the patient with severe osteomyelitis associated with long COVID-19 did not obtain positive results even after HBOT, showing high levels of oxidative stress (ROS, lipid peroxidation, DNA damage) and inflammation biomarkers associated with low levels of antioxidant capacity and nitric oxide metabolites.

In a study on long COVID-19 in England, Kim and colleagues [73] described the condition’s strong similarities to post-traumatic stress disorder. This could be related to the fatigue state recorded in our subjects, especially in the two young athletes, which could be triggered by an infection occurring during a time of increased mental or physical stress.

Furthermore, as reported in the literature, activated immune–inflammatory and oxidative/nitrosative stress pathways may underpin the somatic symptoms of long COVID-19 due to chronic fatigue syndrome [74]. In support of this, after the complete HBOT protocol, the NRS fatigue decreased from 5.6 to 2.8, considering all of the subjects in our study together. According to several authors, long COVID-19 fatigue might be characterized by altered cortical excitability and neurotransmission [75,76,77]; HBOT might have an effect on some brain excitability and neurotransmission parameters, as some authors have discussed [78,79,80]. Furthermore, some therapeutic approaches, such as nutraceuticals, might improve symptoms through this modulation [80,81] in adjuvant to HBOT. Despite the differences in age, physical fitness, individual susceptibility, and number of HBOT sessions, among the studied subjects, the responses were pretty homogeneous (except in the patient suffering from osteomyelitis associated with long COVID-19).

Limitations: The current study has some limitations, namely its small sample size, the heterogeneity among the subjects’ characteristics, and the absence of a pre-COVID-19 evaluation. Therefore, further verification in additional studies is necessary.

## 5. Conclusions

From this preliminary observation, HBOT could be considered a potential treatment for long COVID-19 patients. These results should spur on clinicians to increase treatment samples so that they potentially include other post-viral syndromes in which heterogeneous symptoms (i.e., fatigue and dyspnea) are present.

## Figures and Tables

**Table 1:** Anthropometric and physiological parameters of all subjects. Parameters collected before HBOT. BMI: Body Mass Index; HR: Heart Rate; SBP: Systolic Blood Pressure; DBP: Diastolic Blood Pressure; T: Tympanic Temperature; ISSHL: Idiopathic Sudden Sensorineural Hearing Loss.

Features of the Subjects					
	Athlete 1	Athlete 2	Patient 1 ISSHL	Patient 2 ISSHL	Patient 3 Osteomyelitis
Sex	Male	Male	Male	Female	Female
Age (years)	28	28	48	55	47
Weight (kg)	74	76	82	53	54
Height (cm)	177	181	176	164	163
BMI	23.6	23.2	26.5	19.7	20.3
HR (bpm)	62	60	88	72	75
SBP (mmHg)	110	120	120	125	120
DBP (mmHg)	80	75	80	85	80
T (°C)	36.3	36.5	36.7	37.6	37.8

**Table 2:** Characteristics and symptoms of subjects with long COVID-19. In three cases, an association with another pathology was found (ISSHL and osteomyelitis).

Subject	Duration of Symptoms	Symptoms Long-COVID-19	Categories by Type
1—Athlete	3 to >6 months	Extreme fatigue Dyspnea	Type 3B
2—Athlete	3 to >6 months	Fatigue Dyspnea	Type 3B
ISSHL	3 to >6 months	Fatigue Dyspnea Dry Cough Fever	Type 3B
ISSHL	1 to >3 months	Dyspnea	Type 4A
Osteomyelitis	1 to >3 months	Fatigue Dyspnea Fever	Type 4A

## Data Availability

The data used in this study are presented in the article.

## Abbreviations

ATA	Atmosphere absolute
BMI	Body Mass Index
DBP	Diastolic Blood Pressure
EPR	Electron Paramagnetic Resonance
HBOT	Hyperbaric oxygen therapy
HPLC	High-pressure Liquid Chromatography
HR	Heart Rate
IL-1β	Interleukin-1 beta
IL-6	Interleukin-6
ISSHL	Idiopathic Sudden Sensorineural Hearing Loss
NO	Nitric Oxide
NOx	Nitric Oxide metabolites
OxS	Oxidative Stress
SBP	Systolic Blood Pressure
ROS	Reactive Oxygen Species
TAC	Total Antioxidant Capacity
TQR	Total Quality of Recovery
TNFα	Tumor Necrosis Factor-alpha
VAS	Visual Analog Scale
8-iso-PGF2α	8-isoprostane-PGF2 alpha
8-OH-dG	8-Hydroxy-2′-deoxyguanosine
